# Prevalence, associated factors and predictors of anxiety: a community survey in Selangor, Malaysia

**DOI:** 10.1186/s12888-015-0648-x

**Published:** 2015-10-24

**Authors:** Siti Fatimah Kader Maideen, Sherina Mohd Sidik, Lekhraj Rampal, Firdaus Mukhtar

**Affiliations:** 1 Department of Psychiatry, Faculty of Medicine & Health Sciences, Universiti Putra Malaysia, UPM, 43400 Serdang, Selangor Malaysia; 2 Department of Community Health, Faculty of Medicine & Health Sciences, Universiti Putra Malaysia, Serdang, Selangor Malaysia

**Keywords:** Anxiety, GAD-7, Prevalence, Predictors, Associated-factors, Adults, Malaysia

## Abstract

**Background:**

Anxiety is the most common mental health disorders in the general population. This study aimed to determine the prevalence of anxiety, its associated factors and the predictors of anxiety among adults in the community of Selangor, Malaysia.

**Methods:**

A cross sectional study was carried out in three districts in Selangor, Malaysia. The inclusion criteria of this study were Malaysian citizens, adults aged 18 years and above, and living in the selected living quarters based on the list provided by the Department of Statistics Malaysia (DOS). Participants completed a set of questionnaires, including the validated Malay version of Generalized Anxiety Disorder 7 (GAD 7) to detect anxiety.

**Results:**

Of the 2512 participants who were approached, 1556 of them participated in the study (61.90 %). Based on the cut-off point of 8 and above in the GAD-7, the prevalence of anxiety was 8.2 %. Based on the initial multiple logistic regression analysis, the predictors of anxiety were depression, serious problems at work, domestic violence and high perceived stress. When reanalyzed again after removing depression, low self-esteem and high perceived stress, six predictors that were identified are cancer, serious problems at work, domestic violence, unhappy relationship with family, non-organizational religious activity and intrinsic religiosity.

**Conclusion:**

This study reports the prevalence of anxiety among adults in the community of Selangor, Malaysia and also the magnitude of the associations between various factors and anxiety.

## Background

Anxiety is the most common mental health disorders in the general population, with an early age onset [1]. Anxiety can be characterized by the feelings of tension and worry thoughts. It is a normal process in life. However, if the anxiety becomes severe or start to impair one’s life functioning, it can be categorized as a disorder or illness [2]. Anxiety disorder is a class of mental disorders with differing symptom severity and disability [3], besides being associated with significant societal and economic burden [1]. Types of anxiety disorders include separation anxiety disorder, selective mutism, specific phobia, social phobia, panic disorder, agoraphobia and generalized anxiety disorder (GAD) [4].

Globally, it was estimated that about 272.2 million people had an anxiety disorder at any point in time in 2010 [5]. The point prevalence of anxiety disorders was almost double in females (5.2 %) as compared to males (2.8 %). Adults aged 20–64 years were shown to have the highest prevalence (5.0 %) among the other age groups. Data from the Global Burden of Disease Study 2010 which included 21 regions worldwide, showed the adjusted point prevalence of anxiety disorders of 6.1 % in North Africa/ Middle East and the least in East Asia (2.1 %). It was observed that regions with conflict countries, high income countries and Latin America countries have higher prevalence of anxiety disorders.

In Europe, mental disorders affect about 38.2 % of the population, with anxiety disorders being the most common class of disorder with 14.0 %, which affects 69.1 million of Europe population [6]. The overall 12-month prevalence of anxiety disorders was 14.0 %, with specific phobia the most common with a 12-month prevalence of 6.4 % and panic disorder the least common with 1.8 %.

In another large community survey, the World Health Organization’s (WHO) World Mental Health (WMH) Survey Initiative which included data from seventeen countries involving 85,052 respondents, found the median lifetime prevalence of anxiety disorders of 14.3 %, and 12-month prevalence of 8.3 %,, using the WHO Composite International Diagnostic Interview (CIDI) [7]. The lowest prevalence of anxiety disorder was found in China (lifetime: 4.8 %, 12-month: 3.0 %) and the highest prevalence was in United States (lifetime: 31.0 %, 12-month: 19.0 %). It has been noted that western developed countries have higher prevalence of anxiety disorders compared to the developing countries.

In United States, the National Comorbidity Survey Replication (NCS-R) study using the WHO WMH Survey Initiative version of the CIDI (WMH-CIDI) found a 12-months prevalence of anxiety of 18.1 % among adults in the community [8]. Specific phobia was the most common with a prevalence of 8.7 %, while agoraphobia without panic disorder was the least common, with a prevalence of 0.8 %.

Ruscio AM et al. who analyzed data from the same study (NCS-R) found a lifetime and 12-months prevalence of OCD of 2.3 % and 1.2 % [9]. Data which was analyzed using the Part II NCS-R consisting of 5692 adults in the community showed 12-month prevalence of GAD of 2.7 %, panic disorder (3.7 %), PTSD (3.6 %), social phobia (7.2 %) and specific phobia (9.2 %) [10].

The Australian National Survey of Mental Health and Wellbeing (NSMHWB) conducted among adults in the general population aged 16–85 years showed a lifetime and 12-months prevalence of any anxiety disorder of 20.0 % and 11.8 % [11]. Mclean et al. who examined data from the Collaborative Psychiatric Epidemiology Studies (CPES) involving 20,013 adults in United States found a higher prevalence of anxiety disorders among women as compared to men [12] . The lifetime and 12-months prevalence of any anxiety disorder was 33.3 % and 22.7 % among women, as compared to 22.0 % and 13.0 % in men. Women with anxiety disorders were found to be associated with another type of anxiety disorder and with greater illness burden.

A national survey among adult residents in Singapore showed a lifetime and 12-months prevalence of GAD at 0.9 % and 0.4 % respectively; whereas for OCD the prevalence was at 3.0 % and 1.4 %, respectively [13]. The study used CIDI as the diagnostic tool to assess mental disorders. Unemployment was the only predictor for GAD in Chong et al’s study. For OCD, adults aged 65 and above, divorces/separated, primary education level and chronic illness predicted OCD.

In Malaysia, the most recent national survey in 2011, the National Health Morbidity Survey IV (NHMS IV) which was conducted among adults aged 16 years and above in the community, reported that the prevalence of GAD was 1.7 % [14]. Females, younger age group (16–24 years old) and the Indian ethnic group had higher prevalence of GAD. The prevalence of GAD was also higher among singles, widows/widowers, divorcees and those with tertiary education level. The NHMS IV used Mini International Neuropsychiatry Interview (MINI) as a diagnostic instrument to diagnose anxiety.

Despite the variation in the prevalence of anxiety disorders, it is noted that anxiety disorders affects mental well-being of the population [15] besides significant impairment in quality of life [16]. Studies showed that women had higher prevalence of anxiety as compared to men [3, 17–21]. Anxiety disorders were also found to be more common among individuals with family history of major depressive disorder, disturbed family environment, childhood sexual abuse, low self-esteem, and among those with low education level [19]. Heavy alcohol consumption, smoking, lower socio-economic status, previous use of psychotherapy, previous diagnoses of psychological disorder, history of addiction problem and gastrointestinal disorders were found to be associated with anxiety [22]. Anxiety was also associated with chronic diseases, smoking, physical inactivity, obesity and heavy drinking [23]. Being unmarried, unemployed and having a low social status also increased the risk of mental disorders [17].

Though certain factors have been found to be associated with anxiety, none of these studies were from Malaysia. Cultural and socio-demographic differences may vary from one population to another. Besides that, we were also unable to find any publications on potential factors which might affect anxiety in general population in Selangor. There are no studies that are conducted in the community setting in Malaysia that examine the predictors of anxiety among the adult population. The importance of determining predictors of anxiety is to develop focused community mental health interventions which are effective. The aim of this study was to determine the prevalence of anxiety, its associated factors and the predictors of anxiety among adults in the community of Selangor, Malaysia.

## Methods

### Background of study location

Selangor is the most developed and urbanized state in Malaysia [24]. The Population and Housing Census of Malaysia 2010 showed Selangor as the most populated state in Malaysia, constituting of 5.46 million people. The major ethnic group in Malaysia is the Bumiputera (67.4 %), followed by Chinese (24.6 %), Indians (7.3 %) and Others (0.7 %). The Household Income Survey (HIS) 2012 showed that the mean monthly household income of Malaysian citizens was RM 5000 in 2012 [25].

### Data collection

A cross sectional study was carried out among adults in the community of three districts in Selangor, which were Hulu Langat, Sepang and Klang from 11^th^ June to 30^th^ December 2012. The sampling frame, maps and Enumeration Blocks (EBs) of the living quarters (LQ) in this study were obtained from the Department of Statistics Malaysia (DOS). The study population in this study represents the Malaysian citizens in Selangor as three out of nine districts were selected and the total population for these three districts accounts 2.2 Million of population, which is almost 50 % of the total population of Selangor which consist of 5.4 Million population. The sampling of the households was done by the Department of Statistics Malaysia (DOS), taking into account of the total population of people staying in each of these districts. The total number of Enumeration Blocks and Living Quarters (LQ) that was selected varies according to districts. The allocation of EB in each district was done proportional to the population size in the particular districts. In each EB, eight LQs were selected and in each LQ, two participants were selected. The total number of LQs selected by the DOS in Hulu Langat was 672, followed by Klang (480 LQ) and Sepang 104 LQ. The inclusion criteria were Malaysian citizens, adults aged 18 years and above, and staying in the selected living quarters. The major ethnicity in Malaysia is Malay, Chinese and Indian who lived in the respective households in each of the districts. Those who were unable to comprehend either the Malay or English language were excluded from the study. Data collection was carried out by the main researcher with the assistance from a group of trained research assistants (RAs). A brief introduction about the study, as well as the purpose and benefits of taking part in the study were detailed in the information sheet and all these information were explained to the participants. Informed verbal consent from the participants was obtained prior to the administration of the questionnaire. They completed a set of self-administered questionnaires and were assisted when necessary. The full description on the methodology of this study is detailed in another publication as they were based on the same study [26]. Ethics approval was obtained from the Medical Research Ethics Committee of the Faculty of Medicine and Health Sciences, University Putra Malaysia.

### Instruments

The questionnaire for this study comprised of items on socio-demography, chronic diseases, and history of mental health disorders, depression, stressful life events, perceived stress, domestic violence, self-esteem and religiosity. The questionnaire was in both Malay and English languages, and had been pre-tested in another location not included in this study.

Basic socio-demography variables, such as age, gender, ethnicity, religion, marital status, education level and employment status were assessed in the study. The education level of the participants was categorized into three categories: Primary (Year 1 to Year 6), Secondary (Form 1 to Form 5) and Tertiary (Matriculation/Diploma/Degree/Masters/PhD). The employment status was categorized into three categories: Employed, Unemployed and Pension. The Generalized Anxiety Disorder-7 (GAD-7) was used in this study to determine the presence of anxiety. It is a version of PRIME-MD diagnostic instrument for common mental health disorders and based on the criteria from DSM-IV. The GAD-7 measures generalized anxiety disorder, social anxiety, panic disorder and post-traumatic stress disorder. It consists of 7 items, with each item scored from 0 to 3, with total scores ranging from 0 to 21. A cut-off point of 8 and above on the GAD-7 was used to determine the presence of anxiety in this study as it was demonstrated to have good reliability (sensitivity of 92 % and specificity of 76 %) [27] besides a recommended cut-off score suggested by Kroenke K et al. [28]. The GAD-7 was first developed and validated by Spitzer et al. among patients attending primary care clinics [27]. The Malay version of GAD-7 was validated in the primary care setting by Sherina et al. It has good sensitivity, specificity, concurrent and convergent validity [29]. The GAD-7 was also demonstrated to be a reliable and valid tool to measure anxiety in the general population [30]. The questionnaire of this study was pilot-tested among 250 respondents (249 responded) in another district in Selangor. The GAD-7 was found to have good reliability (Standardized Cronbach’s Alpha = 0.892 and mean inter-item correlation of 0.541). Hence, the findings from this study can be generalized to the general population.

Presence of chronic diseases such as heart disease, diabetes, cancer, arthritis and stroke were self-reported by the participants, based on the diagnosis by doctors or medical professionals. Similarly, the history of mental health disorders were also self-reported by the participants, based on the diagnosis made by the doctors or medical professional.

The Patient Health Questionnaire 9 (PHQ-9) measures the depressive symptoms, based on the criteria from DSM-IV. The PHQ-9 was first developed and validated by Kroenke et al. [31]. The instrument is comprised of nine items, with each item scored from 0 to 3, with total scores ranging from 0 to 27. Participants who had five and above out of nine symptoms in the PHQ-9 in the past two weeks was diagnosed as having major depression. Whereas those who had 2, 3, or 4 symptoms was diagnosed as having other depression. In general, participants who scored 10 and above in the PHQ-9 was categorized as having depression. The Malay version of PHQ-9 was validated in the primary care setting by Sherina et al. [32].

For the assessment of stressful life events, seventeen events associated with depression and anxiety were chosen from Kendler et al. [33]. History of being assaulted, suffering from a serious illness, being abused during childhood and having unhappy relationships with spouse, children and family were among the events assessed in this questionnaire.

The Perceived Stress Scale was developed and validated by Cohen et al. [34]. The PSS-10 was used in this study to measure perceived stress. The PSS-10 consists of ten items, with each of the items rated on a five-point Likert scale (never, almost never, sometimes, fairly often and very often), with total scores ranging from 0 to 40. The mean score of the total items was used as the cut-off point to classify low and high perceived stress.

The questionnaires in this study also included items on domestic violence from the HARK questionnaire [35]. HARK is used as an abbreviation for humiliation, afraid, rape and kick. It is made up of four items, which includes questions on emotional, psychological, sexual and physical abuse from current or ex-partners.

For the assessment of self-esteem, the Rosenberg self-esteem scale (RSES) was used. The RSES was developed by Morris Rosenberg [36]. It is comprised often items, which are rated on a four-point scale (strongly agree, agree, disagree and strongly disagree), with scores ranging from 10 to 40. For the classification of low and high self-esteem, the mean score of the total items was used.

The Duke University Religion Index (DUREL) was used to assess religious involvement [37]. Three major dimensions of religiosity that were assessed in this five-item instrument were: organizational religious activity (ORA), non-organization religious activity (NORA) and intrinsic religiosity (IR).

### Statistical Analysis

Data entry and analysis was done using the IBM SPSS Statistics version 21.0. There were varied numbers of missing data in the questionnaire, depending on the study instrument. Missing data for only that particular instrument were excluded from the analysis. Age was expressed in mean ± standard deviation (SD). The association between the independent categorical variables and anxiety were analyzed using either the Chi square or Fisher’s exact test. The *t*-test was used to determine the differences between the continuous independent variable with anxiety. All the independent variables with p-value < 0.25 on the chi-square and *t*-test were selected for further analysis. Multivariate logistic regression analysis using the Enter method was performed to determine the predictors of anxiety. The predictors of anxiety in this study were selected based on p < 0.05.

## Results

### Response rate

Out of 2512 participants that were approached in this study, 1556 of them participated, giving an overall response rate of 61.90 %. The rest of them refused to participate due to busy and tight schedules, being unwell, unsuitable time of data collection and not interested to participate in the study.

A total of 1455 out of 1556 participants completed the whole items in the GAD-7 questionnaire (response rate of 93.5 %). A-hundred-and-one questionnaires were excluded from the data analysis due to incomplete data. As the number of incomplete data varied from one instrument to another, data were analyzed based on the complete information for the specific instrument.

### Prevalence of anxiety

Based on the cut-off point of 8 and above on the GAD-7, the prevalence of anxiety among adults in the community was 8.2 %. A total of 119 participants had anxiety in this study.

### Socio-demographic characteristics

The age of the participants ranged from 18 to 87 years, with a mean age of 35.36 ± 13.77 years. Anxiety was found to be higher among females (8.4 %) as compared to males (7.7 %). The prevalence of anxiety in the other ethnic groups (9.1 %) was higher compared to Malays (8.4 %), Chinese (8.1 %) and Indians (7.4 %). Other ethnic groups included the natives; which were Bidayu, Iban, Dusun, Kadazan, Melanau and Murut. However, there were no significant associations between age, gender and ethnicity with anxiety. Among the socio-demographic variables, only marital status was significantly associated with anxiety. Divorcees were found to have highest prevalence of anxiety (42.0 %), followed by separated couples (33.3 %), widowed (17.3 %), single (9.3 %) and finally married couples (6.6 %). Participants with primary education level and who were unemployed had higher prevalence of anxiety. Table 1 shows the association between socio demographic profile and anxiety.

**Table 1:** Association of socio demographic profiles with anxiety among participants

Profile of the participants	Presence of anxiety	Absence of anxiety	Total (*n*)	Chi square value	df	*p*-value
	(GAD7 ≥ 8)	(GAD7 < 8)				
	*n* (%)	*n* (%)				
Gender (*n* = 1455)						
Male	42 (7.7)	501 (92.3)	543	0.227	1	0.634
Female	77 (8.4)	835 (91.6)	912			
Ethnicity (*n* = 1455)						
Malay	85 (8.4)	921 (91.6)	1006	0.534	3	0.911
Chinese	10 (8.1)	113 (91.9)	123			
Indian	21 (7.2)	272 (92.8)	293			
Others	3 (9.1)	30 (90.9)	33			
Marital status (*n* = 1455)						
Single	47 (9.3)	458 (90.7)	505	23.148	4	**0.001
Married	59 (6.6)	829 (93.4)	888			
Widowed	9 (17.3)	43 (82.7)	52			
Divorced	3 (42.9)	4 (57.1)	7			
Separated	1 (33.3)	2 (66.7)	3			
Education level (*n* = 1437)						
Primary education	12 (9.8)	111 (90.2)	123	0.676	2	0.713
Secondary education	54 (7.6)	656 (92.4)	710			
Tertiary education	49 (8.1)	555 (91.9)	604			
Employment status (*n* = 1431)						
Employed	54 (7.3)	682 (92.7)	736	0.933	2	0.627
Unemployed	54 (8.6)	577 (91.4)	631			
Pension	4 (6.3)	60 (93.8)	64			

*significant at *p* < 0.05 **significant at *p* < 0.001

### Factors associated with anxiety

Approximately 13 % of the participants with chronic diseases were found to have anxiety. Stroke, arthritis and cancer were the only chronic diseases that were associated with anxiety. Anxiety was also found to be more prevalent among those with personal and family history of mental health disorders. Physical disability was also significantly associated with anxiety. The results are presented in Table 2.

**Table 2:** Association of chronic diseases and history of mental health problem with anxiety among participants (*n* = 1455)

Medical history	Presence of anxiety	Absence of anxiety	Total	OR (95 % CI)	*p*-value
	(GAD-7 ≥ 8)	(GAD-7 < 8)			
	*n* (%)	*n* (%)			
Presence of chronic diseases					
Yes	45 (12.6)	313 (87.4)	358	1.863 (1.312-2.646)	**0.001
No	74 (6.7)	1023 (93.3)	1097		
Heart disease					
Yes	3 (10.0)	27 (90.0)	30	1.228 (0.414-3.645)	0.731
No	116 (8.1)	1309 (91.9)	1425		
Diabetes					
Yes	8 (7.0)	106 (93.0)	114	0.848 (0.425-1.693)	0.637
No	111 (8.3)	1230 (91.7)	1341		
Stroke					
Yes	3 (30.0)	7 (70.0)	10	3.737 (1.427-9.787)	*0.042
No	116 (8.0)	1329 (92.0)	1445		
Hypertension					
Yes	20 (11.8)	150 (88.2)	170	1.527 (0.971-2.402)	0.069
No	99 (7.7)	1186 (92.3)	1285		
Arthritis					
Yes	8 (21.6)	29 (78.4)	37	2.762 (1.458-5.233)	**0.008
No	111 (7.8)	1307 (92.2)	1418		
Cancer					
Yes	3 (50.0)	3 (50.0)	6	6.246 (2.754-14.166)	**0.009
No	116 (8.0)	1333 (92.0)	1449		
Asthma					
Yes	11 (13.3)	72 (86.7)	83	1.684 (0.943-3.005)	0.082
No	108 (7.9)	1264 (92.1)	1372		
Kidney failure					
Yes	0 (0.0)	2 (100.0)	2	Not calculated	1.000
No	119 (8.2)	1334 (91.8)	1453		
Thyroidism					
Yes	2 (22.2)	7 (77.8)	9	2.746 (0.799-9.439)	0.164
No	117 (8.1)	1329 (91.9)	1446		
History of mental health problems					
Yes	13 (43.3)	17 (56.7)	30	5.825 (3.721-9.121)	**0.001
No	106 (7.4)	1319 (92.6)	1425		
Family history of mental health problems					
Yes	10 (22.7)	34 (77.3)	44	2.942 (1.657-5.223)	**0.002
No	109 (7.7)	1302 (92.3)	1411		
Disability					
Yes	8 (53.3)	7 (46.7)	15	6.919 (4.171-11.476)	**0.001
No	111 (7.7)	1329 (92.3)	1440		

*significant at *p* < 0.05 **significant at *p* < 0.001

Table 3 displays the association between stressful life events and anxiety. Fourteen out of seventeen stressful life events were significantly associated with anxiety. Unhappy relationship with children, family, unhappy with work and unhappy relationship with spouse were the strongest events that caused anxiety among participants in this study.

**Table 3:** Association of stressful life events with anxiety among participants (*n* = 1455)

	Presence of anxiety	Absence of anxiety	Total	OR (95 % CI)	*p*-value
	(GAD-7 ≥ 8)	(GAD-7 < 8)			
	*n* (%)	*n* (%)			
Attacked					
Yes	22 (14.7)	128 (85.3)	150	1.973 (1.282–3.036)	**0.002
No	97 (7.4)	1208 (92.6)	1305		
Serious illness					
Yes	19 (16.0)	100 (84.0)	119	2.133 (1.356–3.356)	**0.001
No	100 (7.5)	1236 (92.5)	1336		
Abused during childhood					
Yes	9 (25.7)	26 (74.3)	35	3.319 (1.838–5.994)	**0.001
No	110 (7.7)	1310 (92.3)	1420		
Serious injury due to accident					
Yes	15 (8.9)	153 (91.1)	168	1.105 (0.659–1.853)	0.706
No	104 (8.1)	1183 (91.9)	1287		
Being an orphan before age 10					
Yes	13 (11.0)	105 (89.0)	118	1.390 (0.806–2.395)	0.241
No	106 (7.9)	1231 (92.1)	1337		
Loss of someone very close					
Yes	27 (8.4)	294 (91.6)	321	1.037 (0.688–1.563)	0.863
No	92 (8.1)	1042 (91.9)	1134		
Serious marital problem (*n* = 888)					
Yes	6 (15.8)	32 (84.2)	38	2.532 (1.162–5.520)	*0.035
No	53 (6.2)	797 (93.8)	850		
Serious family problem					
Yes	20 (29.0)	49 (71.0)	69	4.058 (2.679–6.147)	**0.001
No	99 (7.1)	1287 (92.9)	1386		
Serious financial constraint					
Yes	33 (18.4)	146 (81.6)	179	2.735 (1.890–3.958)	** < 0.001
No	86 (6.7)	1190 (93.3)	1276		
Serious housing problems					
Yes	20 (32.3)	42 (67.7)	62	4.539 (3.019–6.823)	**0.001
No	99 (7.1)	1294 (92.9)	1393		
Serious problems at work (*n* = 736)					
Yes	13 (22.8)	44 (77.2)	57	3.777 (2.153–6.627)	**0.001
No	41 (6.0)	638 (94.0)	679		
Lost job					
Yes	11 (21.2)	41 (78.8)	52	2.748 (1.577–4.788)	*0.002
No	108 (7.7)	1295 (92.3)	1403		
Legal problems					
Yes	6 (33.3)	12 (66.7)	18	4.239 (2.154–8.341)	*0.002
No	113 (7.9)	1324 (92.1)	1437		
Relationship with spouse/partner (*n* = 980)					
Unhappy	34 (30.9)	76 (69.1)	110	5.721 (3.858–8.486)	*0.001
Happy	47 (5.4)	823 (94.6)	870		
Relationship with children (*n* = 870)					
Unhappy	20 (32.8)	41 (67.2)	61	6.631 (4.147–10.604)	**0.001
Happy	40 (4.9)	769 (95.1)	809		
Relationship with family					
Unhappy	46 (34.1)	89 (65.9)	135	6.161 (4.458–8.516)	**0.001
Happy	73 (5.5)	1247 (94.5)	1320		
Relationship with work (*n* = 687)					
Unhappy	19 (29.7)	45 (70.3)	64	6.135 (3.670–10.256)	**0.001
Happy	30 (4.8)	590 (95.2)	620		

*significant at *p* < 0.05 **significant at *p* < 0.001

The comorbidity between depression and anxiety in this study was 53.3 % (*n* = 80). There was a significant association between depression and anxiety (OR = 17.832, 95 % CI: 12.649- 25.140), p < 0.001). Participants with depression have almost 18 times higher risk of developing anxiety as compared those who were not.

Domestic violence was significantly associated with anxiety. Among the ever married couples, 48.8 % of adults who experienced domestic violence had anxiety. Among these, 5.5 % (*n* = 36) were women and 1.6 % (*n* = 5) were men. Couples who were abused had almost 9 fold increased risk of suffering from anxiety as compared to those who were not abused (OR = 8.527, 95 % CI: 5.660-12.848, P < 0.001). The most common type of domestic violence were being kicked, hit, or physically abused (53.3 %), followed by being humiliated or emotionally abused (45.5 %), being afraid of their partner (28.6 %) and being raped or sexually abused (20.0 %).

Anxiety was also significantly associated with perceived stress in our study. Based on the mean cut-off point of 15.28 to categorize high and low perceived stress, 13.1 % of adults with high perceived stress had anxiety. There was a significant association between high perceived stress and anxiety (OR = 5.426, 95 % CI: 3.237- 9.094, p < 0.001). The odds of developing anxiety were 5.4 times higher among participants with high perceived stress as compared to those with low perceived stress.

About 14 % of the participants with low self-esteem had anxiety. Self-esteem was significantly associated with anxiety (OR = 4.315, 95 % CI: 2.829-6.583, p < 0.001). Participants with low self-esteem were 4.3 times prone to develop anxiety, as compared among those with high self-esteem. Significant differences were also found between anxiety and organizational religious activity (p = 0.002), non-organization religious activity (p < 0.001) and intrinsic religiosity (<0.001).

### Predictors of anxiety

Thirty-two factors which were found to be associated with anxiety at a p-value of <0.25 were further analyzed using the multiple logistic regression model to determine the predictors of anxiety. Out of these thirty-two factors, four factors were found to be the predictors of anxiety. The predictors arranged from the highest to the lowest risk were depression, serious problems at work, domestic violence and high perceived stress (Table 4).

**Table 4:** Predictors of anxiety based on multivariate logistic regression analysis

Predictors	B	Wald	*p*-value	AOR	95 % CI
Depression	2.868	92.131	<0.001	17.604	9.801–31.619
Serious problems at work	1.745	13.021	<0.001	5.724	2.219–14.767
Domestic violence	1.061	5.486	0.019	2.888	1.189–7.016
High perceived stress	1.020	7.887	0.005	2.772	1.361–5.648
Constant	−10.907	17.128	0.000	0.000	

Analysis was based on a sample size of 1445 participants

After adjusting for other factors in the logistic regression model, depression was found to be the strongest predictor of anxiety. Participants with depression had 17.6 times higher risk of developing anxiety as compared to those without depression. The results also showed that those with serious problems at work were almost six times at higher risk of developing anxiety, as compared to those without this serious problem. The odds of developing anxiety were almost three times higher among participants who experienced domestic violence. It is also showed that those with high perceived stress had three times greater risk of having anxiety, compared to those with low perceived stress.

The variance accounted by the above covariates after controlling for the other confounders best predicts anxiety in our study. However, to avoid over-adjustment when considering the role of other covariates, another multivariate logistic regression analysis was done in which all covariates were considered apart from depression, high perceived stress and low self-esteem (which could be construed as symptoms of anxiety). Based on this analysis, predictors of anxiety (in descending order) were cancer, serious problems at work, domestic violence, unhappy relationship with family, non-organizational religious activity and intrinsic religiosity (Table 5).

**Table 5:** Predictors of anxiety after removing depression, stress and self-esteem

Predictors	B	Wald	*p*-value	AOR	95 % CI
Cancer	2.756	6.172	0.013	15.732	1.789–138.342
Serious problems at work	1.549	14.296	<0.001	4.707	2.109–10.506
Domestic violence	1.376	12.709	<0.001	3.959	1.858–8.437
Unhappy relationship with family	1.135	12.186	<0.001	3.111	1.645–5.884
Non-organizational religious activity	0.287	11.987	0.001	1.332	1.133–1.568
Intrinsic religiosity	0.100	5.257	0.022	1.105	1.015–1.204
Constant	−14.667	36.892	0.000	0.000	

Analysis was based on a sample size of 1445 participants

After removing depression, stress and self-esteem, four additional factors that predict anxiety in this study were found; these were cancer, unhappy relationship with family, non-religious activity and intrinsic religiosity. Cancer found to be the strongest predictor for anxiety. Participants with cancer had almost 16 times higher risk of developing anxiety as compared to those without cancer. Those who were unhappy with their family were 3.1 times more likely to develop anxiety. The odds of having anxiety increased by 1.33 times for every unit increase in non-organizational religiosity activity score. Similarly, the odds of having anxiety increased by 1.1 times for every unit increase in intrinsic religiosity score.

## Discussion

### Prevalence of anxiety

The results in this study indicate that 8.2 % of the population in Selangor has anxiety, while in the Gutenberg Heart Study among the first 5000 individuals in the community enrolled in the study, aged between 35 and 74 years found a prevalence of GAD of 3.4 %, which is half of the prevalence in this study [15]. Even though both the studies used GAD-7 to measure anxiety, the latter study used the shorter version of GAD-7 and specifically assesses GAD, rather than any anxiety in our study, which could be the reason for the difference in the prevalence.

A higher prevalence was found in a study conducted by Johansson et al. among 1329 adults from the Swedish community [20]. This study which also used the GAD-7 at a cut-off point of 8 and above to determine anxiety showed a clinically significant anxiety of 14.7 %, which was almost double the prevalence of anxiety found in our study. The most probable reason for the difference in prevalence between our study and Johansson et al’s study could be due to cultural differences, as well as the reported evidence that western developed countries have higher prevalence of anxiety disorders compared to the developing countries [1]. Higher prevalence of anxiety was also found in Lebanon. The Lebanese Evaluation of the Burden of Ailments and Needs of the Nation (LEBANON) study which was conducted among 2857 Lebanon adults found a prevalence of anxiety disorders of 16.7 % [38]. The risk of developing first onset of mental disorders was elevated due to the exposure to war events.

Almost similar to the prevalence of anxiety in our study, a study conducted in Qatar among patients attending primary care showed an overall prevalence of anxiety disorders of 10.3 % [18]. Similar prevalence was shown in a study in rural communities in Australia where 7.3 % of the population demonstrated mild anxiety and 3.7 % of them reported moderate to severe anxiety, when assessed using the Hospital Anxiety and Depression Scale for anxiety (HADS-A) [39]. Similar prevalence was also found in Korea whereby the lifetime prevalence of anxiety disorder was found to be 9.1 %. Specific phobia was the most common disorder, whereas social phobia the least. [40]. The Korean Epidemiologic Catchment Area (KECA) conducted among adults aged 18–64 years used the Korean version of CIDI as the diagnostic tool.

A community study in a rural area of the East Coast of Peninsular Malaysia found a slightly higher prevalence of anxiety of 12.90 % as compared to our study [41]. The study used HADS to measure anxiety and depressive symptoms. The slight discrepancies in the prevalence could be due to the different screening instrument used.

Nevertheless, the prevalence of anxiety in our study is still within the range found in other studies, whereby overall prevalence of anxiety disorders were shown to be within 5.6 % to 18.1 % [17], and is also similar to the findings by Baxter et al. in their systematic review and meta regression, which demonstrated a global current prevalence of any anxiety disorders of 7.3 % and past year prevalence of 11.6 % [3]. These results were obtained after the adjustment of methodological differences.

A lower prevalence of anxiety was found in Nigeria. The Nigerian Survey of Mental Health and Well-being which was conducted among 4984 adults in the community found a lifetime prevalence of anxiety disorders of 5.7 % [42]. The low prevalence of anxiety disorders in the study was most probably due to participants who declined to report their symptoms to a person they didn’t know (interviewer) and due to the stigma attached to mental illness.

Even though there is a wide variation in the prevalence of anxiety from one country to the other, it should be acknowledged that methodological differences play an important role in the estimation of prevalence. Prevalence period, diagnostic criteria used, survey instrument, survey administration, study population and response rate could also have affected the prevalence.

### Predictors of anxiety

The strongest predictor of anxiety in this study was depression. The comorbidity between anxiety and depression was 53.3 %. This finding was similar to that found in the study by Johansson et al. and Kessler et al., whereby 57.5 % of depressed individuals were found to have anxiety [20, 43]. In our study, adults who were depressed had almost 19 times higher risk of having anxiety compared to those without depression. Data from the Netherlands Study of Depression and Anxiety (NESDA) showed that adults with comorbidity of anxiety-depression had higher levels of cognitive, physical and social disability [44]. Comorbidity between anxiety and depression was associated with elevated severity of mental illness, functional impairment and medical cost [45].

Serious problems at work were one of the predictors of anxiety in our study. Adults who had serious problems at work had six times higher risk of having anxiety. Melchior et al. demonstrated that when there is no prior history of mental health disorders, work stress precipitates the first onset of depression and anxiety [46]. Anxiety was found to be significantly associated with burnout, with the highest odds in both the high level and intermediate level of burnout [47]. Longitudinal data from the Netherlands Mental Health Survey and Incidence Study (NEMESIS) among 2646 employees aged 18–65 years showed psychological demands (related to work load) predicted the incidence of depressive and anxiety disorders among male and female employees [48]. The data also showed that job insecurity among females was a risk factor for anxiety disorders.

Domestic violence was also one of the factors that predicted anxiety in our study. A study by Ludermir et al. found almost 50 % of women who were abused had developed mental disorders [49]. In another study, it was shown that women who were physically, sexually and psychologically abused had increased risk of developing anxiety [50]. Pregnant women who experienced intimate partner violence (IPV) were found to have higher risk of common perinatal mental disorders (CPMD) of depression and anxiety (CPMD) [51]. The risk of CPMD was greater among those who experienced more than one type of IPV. A study in Tunisia among married women found a prevalence of 33.9 % of anxiety among women who were abused [52]. The study found a significant association between domestic violence and anxiety among married women. Those who were abused were found to have reduced desire, arousal and response disorders and lack of sexual satisfaction.

Perceived stress was also one of the significant predictors of anxiety in our study. Adults with high perceived stress had almost three-fold increased risk of experiencing anxiety as compared to those without this stress. It was observed that adults with anxiety disorders were significantly associated with a higher level of perceived stress as compared to the general community [53]. Adults with severe anxiety were found to be more susceptible for greater stress.

The association between cancer and anxiety has been shown in many studies. This study found cancer to predict anxiety. Participants with cancer had almost 16 times higher risk of having anxiety compared to those without cancer. It has been demonstrated that high prevalence of anxiety was associated with cancer; with specific phobias, panic disorder and GAD being the most common type of anxiety being reported [45]. O’Neill et al. who used the data from the 9 World Mental Health Survey (WMH) that was carried out in 19 countries demonstrated that among the 16 mental disorders that were assessed, after the adjustment for comorbidity, cancer was associated with panic disorder, specific phobia and alcohol abuse [54].

Other than that, unhappy relationship with family was also one of the predictors of anxiety in this study. It is found that adults who were unhappy with their family members had almost three times higher risk of experiencing anxiety as compared to those without this problem. Francis et al. who conducted a study among 397 family caregivers of cancer patients showed that caregivers who have good family bonding had lower anxiety and depressed mood as compared to those with poor family relationship [55]. In another study the authors examined psychosocial risk and protective factors of GAD among adults in United States and found that good family cohesion act as a protective factor against anxiety [56]. The study also showed that negative family interaction predicts anxiety.

Religiosity was also a significant predictor of anxiety in this study. While many studies found religiosity as a protective factor against mental illness such as anxiety and depression [57, 58], the results of our study found intrinsic religiosity and non-organizational religious activity increased the risk of anxiety. Koenig in his review on religion, spirituality and health described in depth on the positive and negative associations between religiosity and anxiety [59]. Religiosity was associated with greater well-being, improved coping skills to manage stress and improved mental health. Even though religiosity increases mental health, there is possibility when it did not. Some used religion as to warrant their hatred, aggression, possessive to gain power, escape from dealing with family problems and presence of stress due to inability to reach high levels of religious standard, could lead to anxiety. In another study, the results from a longitudinal cohort study among adults in Baltimore showed no association between religious worship attendance and spirituality with anxiety [60]. Ellison et al. found an inverse relationship between frequency of religious attendance and belief in afterlife with anxiety [61]. There are mixed and contradictory findings on the association between religiosity and anxiety. Various factors such as instruments used to measure religiosity, sampling method, low sample size, ethnicity, culture and the study population could be the possible reasons for the differences in the relationship. Hence, there is a need for future community studies in the local setting to explore the relationship between religiosity and anxiety using standardized measures and method.

### Strengths

The strength of this study is that it was conducted using the Malay language; which is the national language of the local population. The Malay version was available for each assessment, including the validated Malay version of GAD-7 to detect anxiety. Another strength of the study is the sampling method, where the selection of the EBs and LQs was conducted independently by the DOS, Malaysia.

### Limitations

There could be some limitations that might have occurred during the cross sectional survey. The prevalence of anxiety in this study could have been affected by methodological differences. In addition to that, causality also cannot be inferred due to the nature of the cross-sectional study. The presence of chronic diseases was self-reported. However, the participants were reminded to state it based on the diagnosis made by the doctors or medical professionals during their medical health examination. Recall bias might have occurred, as some of the questionnaires require to state whether the condition had been present for the past two weeks. Nevertheless, the participants were requested to think carefully before answering the questions. We could have missed the ‘true patients’ who were being admitted to the hospitals, or staying in hostels, institutions, or those who were not at home during the data collection. However, this limitation is beyond our control even though we did attempt to visit the households for the second time. Due to these limitations, the findings of this study should be interpreted with caution.

## Conclusion

This study reports the prevalence of anxiety among adults in the community of Selangor, Malaysia and also the magnitude of the associations between various factors and anxiety. The predictors of anxiety in our local population were also identified.
